# A Numerical Model of Blood Flow Velocity Measurement Based on Finger Ring

**DOI:** 10.1155/2018/3916481

**Published:** 2018-10-03

**Authors:** Fu Yusheng, Qiyu Wang, Jianbo Yi, Dan Song, Xin Xiang

**Affiliations:** ^1^School of Information and Communication Engineering, University of Electronic Science and Technology of China, Chengdu, Sichuan 611731, China; ^2^School of Electronic Engineering, University of Electronic Science and Technology of China, Chengdu, Sichuan 611731, China; ^3^Department of Internal Medicine, The Affiliated Hospital of University of Electronic Science and Technology of China, Chengdu, Sichuan 611731, China; ^4^School of Institute of Aeronautics and Astronautics Engineering, Air Force Engineering University, Xi'an, Shaanxi 710051, China

## Abstract

Aiming to measure the blood flow velocity in a finger, a novel noninvasive method, i.e., a ring with a heat source chip and a temperature sensor, is designed in this paper. The heat source chip is used to heat the finger and generate heat diffusion between the chip and the temperature sensor. And the temperature sensor is designed to measure the temperature difference. Since the blood flow is the main medium of heat diffusion in bodies, part from the heat energy in the tissue will be taken away by the flowing blood. Therefore, the blood flow velocity can be acquired via its relationship with the temperature difference. Compared to the ultrasound Doppler method and the laser Doppler method, the proposed method guarantees a more convenient operation in more flexible work sites. We also analyze the theory between heat transfer and laminar flow. Finally, several simulations are conducted, and the influence of the relevant factors (i.e., the number of blood vessels, the radius, etc.) corresponding to the simulation results is also discussed.

## 1. Introduction

Both the hemodynamics and blood rheology are important aspects in the medical research field [1, 2]. The blood flow information measurement is valuable for the cardiovascular diagnosis and some other vascular disease diagnosis. The process in which blood flow reaches the venules through tiny capillaries arterioles is called blood microcirculation. And over 80% substance exchange of a person is completed in microvascular area. The blood microcirculation is present throughout people's bodies. Therefore, the blood flow information on the microcirculation is particularly significant. Researching blood microcirculation is beneficial for understanding the pathogenesis, understanding the analysis, and determining the pathogenetic condition. Among the blood flow information, the blood flow velocity is one of the main parameters to characterize the microcirculation [3–6].

There are several traditional blood flow measurement methods, such as the tracer injection method, the plethysmography method, the electromagnetic flow meter based method, etc [7–11]. Additionally, some of the blood flow sensors and mechanical blood flow meter are also commonly used in biomedical or clinical experiments [12–16]. However, the low spatial resolution of traditional methods cannot meet people's needs any more, in the meantime some methods of them are hard to operate, so the better methods are needed.

Microfluidic technology has emerged as a powerful tool for biological cell manipulation and disease diagnosis [17–32]. With the technology diffusion and technology accumulation, a number of the blood flow velocity measurement methods appeared with benefits of high-resolution, nondestructive, and fast test. These methods also become increasingly important in the biomedical field. Among these methods, the Doppler-based method (based on ultrasound and based on laser) is the most commonly used method of the blood flow velocity measurement. The ultrasound Doppler method (the laser Doppler method) uses ultrasonic (laser) to make the blood images within the organization on the screen, and then blood flow velocity is obtained via testing the change of these blood images [33–37]. At present, for scientific research and diagnosis, most hospitals are equipped with the Doppler anemometry, which has been improved and applied in clinic for decades. Obviously, the Doppler based methods have been recognized by the broad masses of medical workers. However, the Doppler anemometry is too huge to operate conveniently, and it requires operating personnel of a higher level of knowledge. Besides, the test site is relatively fixed. The Doppler anemometry is not suitable for fingers or some other parts of human bodies, and it is not convenient enough in some work sites (i.e., home, scientific research establishments, etc.).

In this paper, a noninvasive method is proposed to measure blood flow velocity of a finger via heat diffusion between a finger and a ring. The ring consists of a heat source chip and a temperature sensing display device. The temperature difference between these two components indicates velocity information, since the blood flow is the main medium of heat diffusion for a person. Based on actual range of parameters, we assume that the related factors (i.e., the number of blood vessels, radius, etc.) are under the constant conditions, and different blood velocities will necessarily cause diverse temperatures which are obtained from temperature sensor. By using the empirical parameters of the blood, the bone, the adipose, and the finger blood flow rate of normal adults, we also analyze the theory between the heat transfer and the laminar flow, since these two theories are both tightly connected with the overall model [38–44]. Finally, we conduct some simulations and give detailed discussions about the simulation results.

## 2. Method and Theory

**Figure 1:**
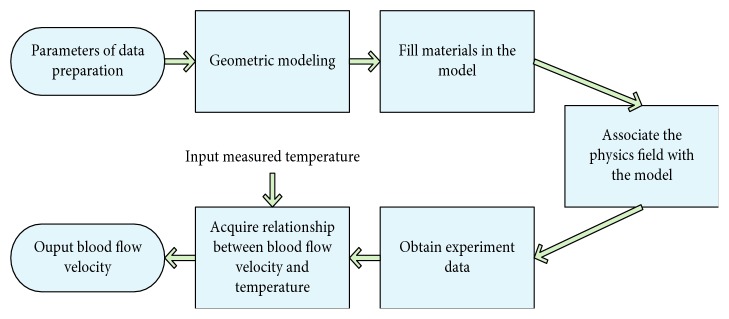
The frame diagram of the proposed method.

The frame diagram of the proposed method is shown in Figure 1. We use multiphysics simulation software—COMSOL Multiphysics to establish the geometric model firstly.

**Figure 2:**
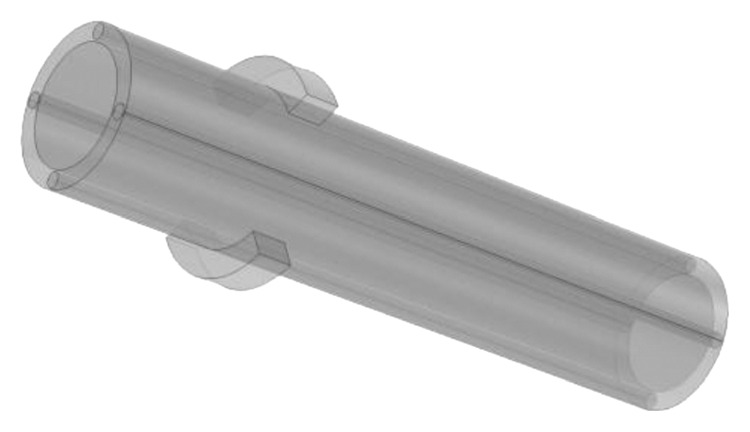
Overall model diagram.

In the human body, three types of blood vessels exist, and any one kind of them has its own function. All these three types of blood vessels can be roughly regarded as tubes with liquid. According to [45–47], in addition to the thumb, the digital arteries are on either side of the other four fingers, and they are relatively fixed and straight. The thickness of the proximal artery is relatively symmetrical. In the early stages of modeling, in order to simplify complexity of the calculation, we blur the conception of these three vessels since they have many similar functions and model the numerous capillaries in fingers as a larger vessel. In modeling, due to the smaller dimension of the blood vessels and especially when they are relative to fingers, we set blood vessels as cylinders. Moreover, since the object we study is a section of a finger, the outline of a section of a finger and the bone can both be roughly modeled as cylinders, even though human fingers are not symmetrical. The same way to model the heat source component and the temperature sensing display device, to establish a preliminary model, we simplify them as 1/4 parts from ring cylinders. The overall model diagram is shown in Figure 2.

After the preliminary model is established, we fill the necessary materials into the model. The needed materials are chiefly 5 types: the blood, fat, the bone, metal, and air. According to [38–42], the parameters corresponding to these materials are shown in Table 1.

**Figure 3:**
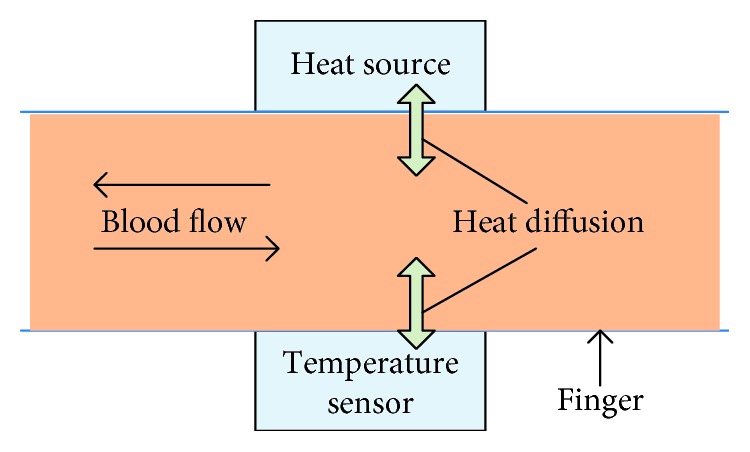
The schematic of heat diffusion in the proposed model.

In the method we proposed, we assume that two main components are considered in the ring as the schematic shown in Figure 3. About the heat source component, we assume that the temperature of a heat source is 323.15 K, which is higher than the human finger temperature since the temperature of human fingers is around 303.15 K [48]. Temperature difference leads to temperature transfer in the media. The heat transfers from the heat source component, through finger and finally gets to the temperature sensing component, where the temperature sensor is sensitive to a tiny temperature change. Because a part from the heat will be taken away by the blood flow, the temperature of sensor will be lower than 323.15 K.

In reality, each person has exclusive state of fingers, and people do not need particularly accurate health data in some conditions. Therefore, the relationships between the blood flow velocity and the obtained temperature can be paid attention in those states. These relationships rely on normal ranges of each parameter in truth. The more situations are considered, the better this presupposed ring works.

In heat transfer process, the connected physics fields are the heat transfer field and the laminar flow field. The heat transfer physics field contains two parts that are the heat transfer in solid and the heat transfer in liquid. And the first part is corresponding to the ring, the adipose, and the bone, while the second part is corresponding to the vessels and the blood.

To simulate the process of heat diffusion in the proposed model, the heat transfer interface (interface 1) and the laminar flow interface (interface 2) of simulation software (COMSOL Multiphysics) are mainly used, which are relied on the theory of heat transfer and the theory of laminar flow [49].

**Figure 4:**
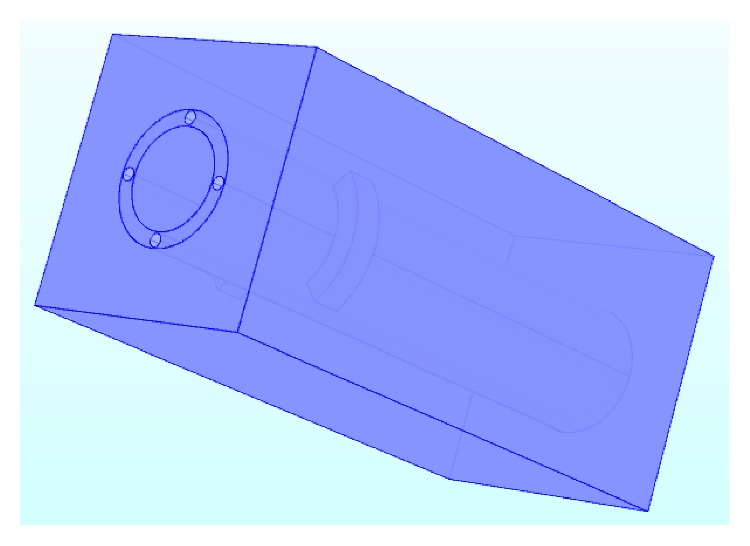
The sketch map of areas connected with interface 1.2 in the proposed model.

Interface 1 contains two parts, the one about fluids is called interface 1.1, and the other one about solids is called interface 1.2 [50–52]. The interface 1.2 is used to model the heat transfer process in solids. The areas connected with interface 1.2 are the air, the metal ring, the adipose, the bone, and the blood vessel, which are shown in Figure 4.

The physics involved in interface 1.2 can be described as a temperature equation which is shown below. The interface is used for solving the following equation:

(1) $$\begin{array}{c} \rho C_{\text{P}}\left(\frac{\partial T}{\partial t}+u_{\text{trans}}\cdot \nabla T\right)+\nabla \cdot \left(q+q_{r}\right)=Q+Q_{\text{ted}}. \end{array}$$

**Figure 5:**
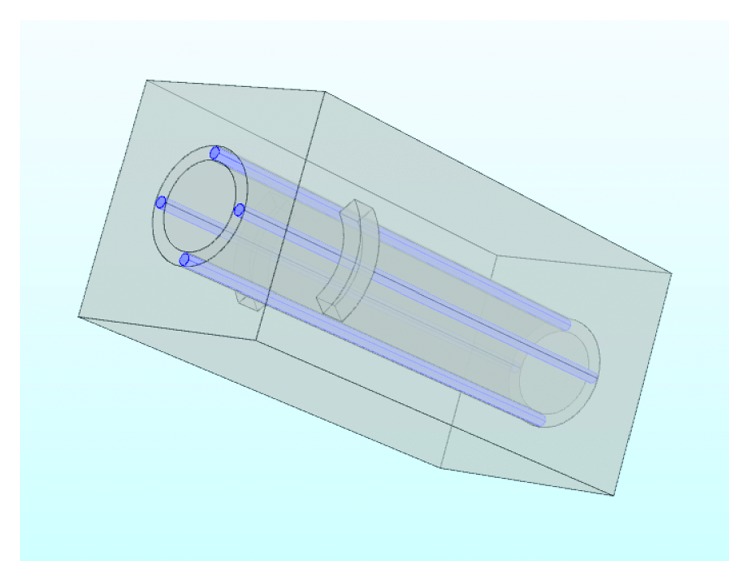
The sketch map of areas connected with interface 1.1 and interface 2 in the proposed model.

The interface 1.1 is used in the area of the blood vessel. And the areas connected with interface 1.1 are shown in Figure 5. The interface is used for solving the following equation:

(2) $$\begin{array}{c} \left\{\begin{array}{c} \rho C_{\text{P}}\left(\frac{\partial T}{\partial t}+u\cdot \nabla T\right)+\nabla \cdot \left(q+q_{r}\right)=Q+Q_{\text{p}}+Q_{\text{vd}},\\ q=-k\nabla T, \end{array}\right. \end{array}$$

where two symbols are used to describe the heat flux: **q** (conduction) and **q**_*r*_ (radiation); *ρ* presents the density; and the thermal conductivity is described as *k*. In equations, the specific heat capacity is presented as *C*_*P*_, and *T* describes the absolute temperature. **u** is the velocity vector, besides, **u**_trans_ presents its translational motion. In Equation (1), two parts are on the right side of the equal sign. The first one means additional heat sources, the second one means the thermoelastic damping and accounts for thermoelastic effects in solids:

(3) $$\begin{array}{c} Q_{\text{ted}}=-\alpha T:\frac{dS}{dt}. \end{array}$$

The *d*(·)/*dt* operator is the material derivative, as described in the time derivative subsection of material and spatial frames.

In Equation (2), three parts are on the right side of the equal sign. The first one means the heat sources except viscous dissipation.

The second one describes the work produced under changed pressure.

(4) $$\begin{array}{c} Q_{\text{p}}=\alpha_{\text{p}}T\left(\frac{\partial p}{\partial t}+u\cdot \nabla p\right), \end{array}$$

where *p* presents the pressure, and the coefficient of thermal expansion is defined as *α*_*p*_:

(5) $$\begin{array}{c} \alpha_{\text{p}}=-\frac{1}{\rho }\frac{\partial \rho }{\partial T}. \end{array}$$

The third one represents the viscous dissipation in the fluid:

(6) $$\begin{array}{c} Q_{\text{vd}}=\tau :\nabla \mathrm{u,} \end{array}$$

where the viscous stress tensor is presented as *τ*. Operation “:” here describes contraction between the tensors, and the details are shown as follows:

(7) $$\begin{array}{c} a:b=\underset{n}{\sum }\underset{m}{\sum }a_{nm}b_{nm}. \end{array}$$

Laminar flow is a kind of single-phase fluid-flow, and interface 2 is to describe some parameters under the circumstance. For instance, the velocity and pressure fields in the laminar flow are determined by the scientific regime. A parameter called Reynolds number can be used to describe the state of the flow, and once this parameter is below a certain critical value, the flow remains laminar [53–56]. When this parameter is higher, disturbances grow and laminar transits to turbulence [57]. In the model, the interface 2 is used in the area of the blood vessel, which is shown in Figure 5.

The physics behind interface 2 can be described below, and these equations are according to Navier–Stokes equations.

(8) $$\begin{array}{c} \frac{\partial \rho }{\partial t}+\nabla \cdot \left(\rho u\right)=0, \end{array}$$

(9) $$\begin{array}{c} \rho \frac{\partial u}{\partial t}+\rho \left(u\cdot \nabla \right)u=\nabla \left[-pI+\tau \right]+F, \end{array}$$

(10) $$\begin{array}{c} \rho C_{\text{p}}\left(\frac{\partial T}{\partial t}+\left(u\cdot \nabla \right)T\right)=-\left(\nabla \cdot q\right)+\tau :S-\frac{T}{\rho }{\left.\frac{\partial \rho }{\partial t}\right|}_{\text{p}}\left(\frac{\partial p}{\partial t}+\left(u\cdot \nabla \right)p\right)+Q, \end{array}$$

where *I* presents the unity tensor, the volume force vector is described as **F**, and the strain-rate tensor is described as **S**:

(11) $$\begin{array}{c} S=\frac{1}{2}\left(\nabla u+{\left(\nabla u\right)}^{T}\right). \end{array}$$

Conservation of mass is involved in Equation (8), and it is a continuity equation.

Conservation of momentum is involved in Equation (9), and it is a vector equation.

Conservation of energy is involved in Equation (10).

In addition, the other parameters are consistent with parameters in heat transfer interface. Thereinto, the velocity vector **u** is the most important parameter that we pay attention to, which represents blood velocity here. In order to reflect the combined effect in this simulation, we blur the conception of the capillary, the vein, and the artery. For the adult group, we choose an appropriate range of blood velocity, which is from 3 cm/s to 12.6 cm/s. It is based on the empirical values of blood velocity, which ranges from 4.9 cm/s to 19 cm/s in arterial while ranges from 1.5 cm/s to 7.1 cm/s in the vein.

The model we establish is a multiphysics model, which contains temperature coupling and flow coupling. About the boundary, the Marangoni effect works here [49]. The boundary condition can be described as the following equation:

(12) $$\begin{array}{c} -\left[\rho I+\mu \left(\nabla u+{\left(\nabla u\right)}^{T}\right)-\frac{2}{3}\mu \left(\nabla \cdot u\right)I\right]n=\gamma \nabla_{t}T. \end{array}$$

In the simulation, the fine degree of the calculation is reasonably considered, which is the model meshing. In the area of the blood vessels, we mesh it with the finest gridding. The other parts are meshed more roughly compared to the blood vessels.

With a series of parameters we set in simulation, we obtain the empirical data (the temperature corresponding to the blood vessel velocity). And with the empirical data, the relationship between the temperature and the blood flow velocity can be characterized as a polynomial. And once we get the polynomial and the temperature observed in reality, the blood flow velocity can be described.

## 3. Simulations and Results

**Figure 6:**
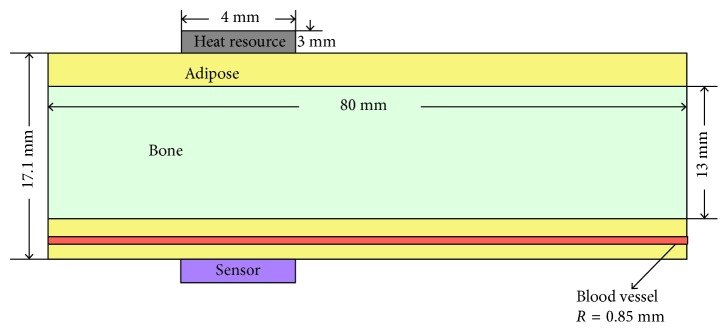
Geometric dimensioning diagram of the model.

The blood vessels in human fingers are diverse, and they hold comparatively great differences on the diameter (800 *μ*m∼1.8 mm). Hence, we choose a suitable range (0.3 mm∼0.45 mm) to describe the blood vessel which is built in the model. In simulation, the physical dimension of model is shown in Figure 6. On the basis of actual size, the radiuses of the three cylinders (the blood vessel, the finger bone, and the geometric shape of finger) are set as 0.4 mm, 0.65 mm, and 0.85 mm, respectively. In the meantime, the lengths of these three cylinders are all set as 80 mm.

**Figure 7:**
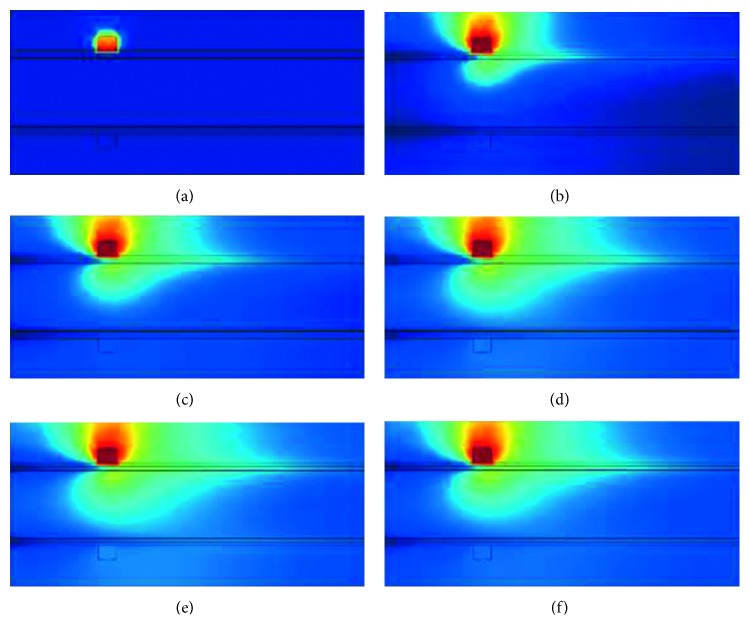
Cross-sectional cutaway view of heat transfer process in a finger. These 6 pictures (a∼f) are arranged according to the chronological order. The warmer the color is, the higher the temperature is.

Based on the operation above, we calculate the model by using gridding. From a cross-sectional cutaway view of one certain simulation, we can visually observe the whole energy flowing process, as shown in Figure 7.  Figure 7(a) shows the initial state, heat energy still gathered at the source. From Figures 7(b) to 7(e), we can visually observe the heat diffusion in the finger. Due to the blood flow, the heat distribution is not symmetrical. Comparing to the Figure 7(e), the warmer scope of the Figure 7(f) is slightly smaller.

**Figure 8:**
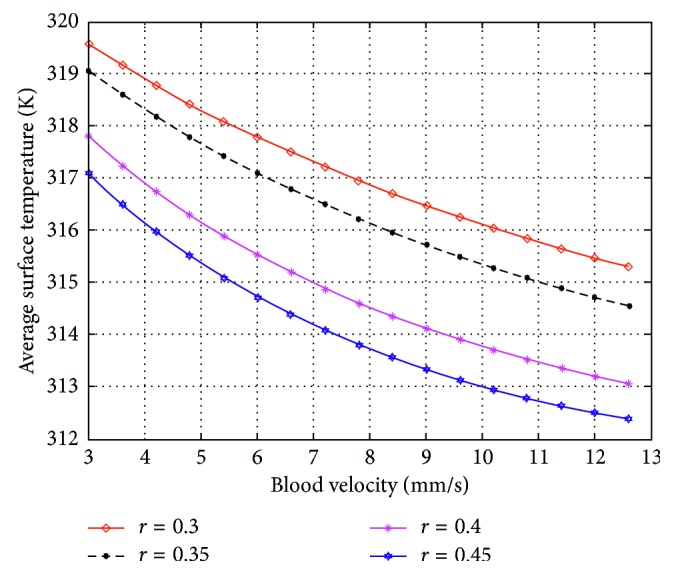
Empirical curves of the blood flow velocities and the temperatures in 4 situations, where only one blood vessel is considered. “*r*=0.3” represents blood vessel's radius, 0.3 mm; “*r*=0.35” represents blood vessel's radius, 0.35 mm; “*r*=0.4” represents blood vessel's radius, 0.4 mm; and “*r*=0.45” represents blood vessel's radius, 0.45 mm.

**Figure 9:**
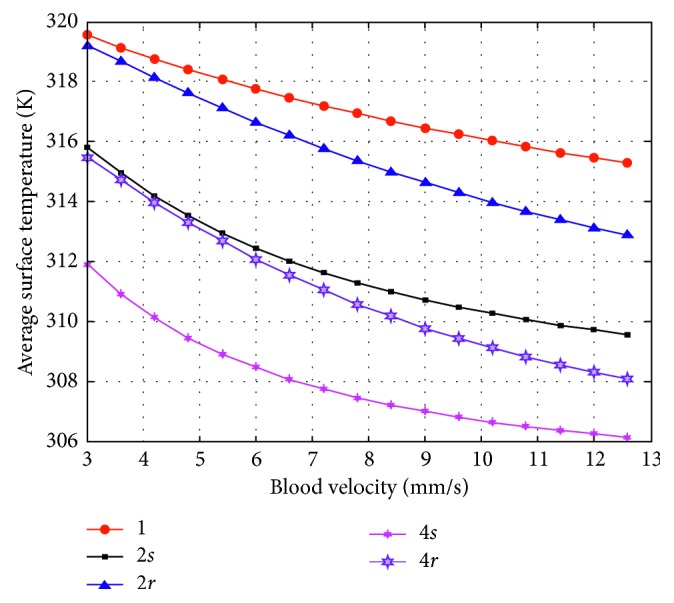
Empirical curves of the blood flow velocities and received temperatures in 5 situations, where the radius of blood vessel is set as 0.3 mm. “1”: one blood vessel is built in the model; “2*s*”: two blood vessels with the same direction of blood flow are built in the model; “2*r*”: two blood vessels with the reverse direction of blood flow are built in the model; “4*s*”: four blood vessels with the same direction of blood flow are built in the model; and “4*r*”: four blood vessels are built in the model where the blood flow directions of two blood vessels are the same while the blood flow directions of other two blood vessels are reverse.

In this simulation, a curve is fitted by using multiple simulation results. Within the reasonable range (3 cm/s∼12.6 cm/s) of the blood flow velocity, we conduct 51 times of simulation to get 51 pairs of results (“velocity-temperature”), where the relevant factors (the blood vessel number, the blood flow direction, and the blood vessel radius) are temporarily fixed. Nevertheless, we aim not only at these 51 pairs of results, but the influence of these 3 factors. Therefore, we change the value of these factors for another several simulations again. Eventually, we obtain the integrated empirical curves which are shown below. Figures 8 and 9 show the experience curves of blood flow velocities and temperatures, and these curves signally reflect the difference between each kind of simulation data, where the data come from several different conditions.

The differences between these empirical curves are discussed, which come from the 3 factors (the blood vessel number, the blood vessel radius, and the blood flow direction).

According to Figure 8, when only one blood vessel is considered, the larger the blood vessel is, the more heat is taken away, and the empirical curve shifts down more. In the meantime, the slopes of these curves are approximative.

According to Figure 9, when the radius of blood vessels is 0.3 mm and the blood flow directions are the same, more blood vessels are considered, more heat is taken away, and the empirical curve shifts down more. In the meantime, the slopes of these curves are approximative.

The other two curves in Figure 9 describe the situations that the blood flow directions are reverse. The curve marked as “2*r*” is upon the curve marked as “4*r*,” and both the two curves decline when blood flow velocity increases, meanwhile, the degrees of inclination of these two curves are similar. Obviously, these two curves indicate that more blood vessels take more heat away. And a higher blood flow velocity brings more heat away too. When the number of blood vessel is the same, the curve (2*r*, 4*r*) under the same blood flow direction is sharper than the curve (2*s*, 4*s*) under the reverse blood flow direction.

In a certain condition, a polynomial can be provided via fitting the corresponding empirical curve, where the relationship between the temperature and the blood velocity in such a condition is described.

**Figure 10:**
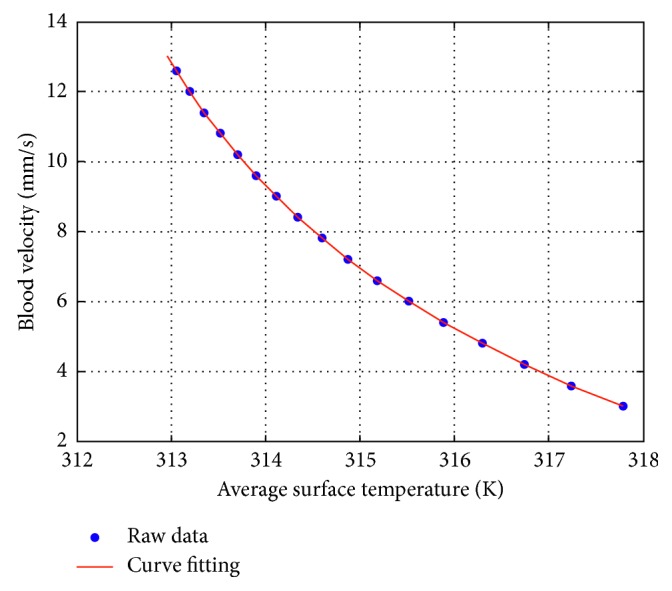
Schematic diagram of fitting curve and data points when only one vessel is considered in the model.

Then, the blood flow velocity can be obtained once the temperature and the corresponding polynomial are established. For instance, when the blood vessel's radius is set to 0.4 mm and only one blood vessel is built in the model, the several pairs of data that are obtained from the simulation are marked as blue points, which are shown in Figure 10. Besides, relying on these data, the fitting curve is marked with red color and shown below. For better fitting performance, the error precision is controlled within 0.00001. Afterwards, the corresponding polynomial is provided and shown in the following equation:

(13) $$\begin{array}{c} f\left(x\right)=0.005x^{2}-0.0817x+5.1749. \end{array}$$

## 4. Conclusion

In this paper, a noninvasive method has been presented for the blood flow velocity measurement. Compared with the Doppler based method, the structure of the method in this paper is simpler. Besides, the ring that came up in the method is smaller than that used in the Doppler anemometry in the market, so the device of this method is easier to carry, and the work sites of this method are more flexible.

By using this method in modeling, we have obtained a schematic diagram of heat transfer process and 10 empirical curves. Relying on these empirical curves, one of the relationships between the blood velocity and the observed temperature has been described with a polynomial. Moreover, the influences of the 3 factors (the blood vessel number, the blood vessel radius, and the blood flow direction) on the empirical curves have been discussed.

Even though the parameters of the model are set according to actual data, the polynomial in this paper is not precise enough due to individual differences. The purpose of this paper is to analyze the relationship according to a relatively primitive model. Refining and optimizing are needed for a more feasible method in later study. For instance, the temperature of the heat source is stable in the primitive model, and different states of the temperature may lead to a better result. Changing the outline of the finger, ring material, and arrangement of blood vessels can make the model more realistic. The diverse group of people is also an important aspect for improving the method. The primitive model will be improved with more reasonable conditions.

## Figures and Tables

**Table 1:** Parameters of materials.

	Blood	Adipose	Bone	Air	Ring
*C*_*p*_ (J/(kg·K))	3300	0.5	1100	1.3	1.2
*k* (W/(m·K))	2503	0.26	911	—	—
*ρ* (kg/m^3^)	2100	0.3	2710	—	—
*μ* (Pa·s)	1025.3	0.024	1.29	—	—
*γ*	385	400	8960	—	—
